# Polycystic Kidney Disease–Related Disease Burden in Adolescents With Autosomal Dominant Polycystic Kidney Disease: An International Qualitative Study

**DOI:** 10.1016/j.xkme.2022.100415

**Published:** 2022-01-21

**Authors:** Dorothee Oberdhan, Franz Schaefer, Jason C. Cole, Andrew C. Palsgrove, Ann Dandurand, Lisa Guay-Woodford

**Affiliations:** 1 Otsuka Pharmaceutical Development & Commercialization, Inc, Rockville, MD; 2 Division of Paediatric Nephrology, Center for Paediatrics and Adolescent Medicine, University of Heidelberg, Heidelberg, Germany; 3 P3 Research Consulting, LLC, Torrance, CA; 4 Center for Translational Research, The Children’s Research Institute, Children’s National Health System, Washington, DC

**Keywords:** Adolescent, autosomal dominant, autosomal dominant polycystic kidney disease, cystic, disease burden, patient-reported outcome, pediatric, polycystic kidney, PRO, QoL, quality of life

## Abstract

**Rationale & Objective:**

Little is known about symptoms and disease impacts in adolescents with autosomal dominant polycystic kidney disease (ADPKD). The objective of the study was to explore these issues from the adolescent patient’s perspective.

**Study Design:**

Observational, qualitative study.

**Setting & Participants:**

Eligible participants were 12-17 years old and had a diagnosis of ADPKD. Semi-structured interviews were conducted in 18 cities in 13 countries to elicit participant experiences of ADPKD-related symptoms and physical, social, and emotional impacts.

**Analytical Approach:**

Interviews were recorded, transcribed, and coded. Symptom and impact frequencies from the interviews were calculated, and representative quotes concerning elicited concepts were collated.

**Results:**

Thirty-three participants (mean age, 14.6 years; 42.4% female) completed interviews. Frequently reported symptoms included urinary urgency (n = 10; 30.3%) and back pain (n = 9; 27.3%). Consistent with previous findings in adults, participants experienced 3 primary types of pain: dull kidney pain, severe or sharp kidney pain, and a feeling of fullness and/or discomfort. Reported disease impacts included avoiding sports and physical activity (n = 10; 30.3%), missing school (n = 6; 18.2%) and social activities (n = 6; 18.2%), and feeling worried (n = 6; 18.2%), sad (n = 4; 12.1%), or frustrated (n = 3; 9.1%) about the disease and their future. Approximately one-fifth of participants (n = 7; 21.2%) reported that they were bothered or impacted by dietary limitations (primarily the need for reduced sodium intake and increased water intake).

**Limitations:**

The study had a small sample size. The researchers were unable to conduct focus groups with participants because of parental preferences.

**Conclusions:**

The findings from this exploratory study indicate that a substantial proportion of adolescents with ADPKD experience physical, social, and emotional impacts from their disease.

**Plain-Language Summary**

A qualitative study was conducted to understand the impact of autosomal dominant polycystic kidney disease (ADPKD) on adolescents from the patient perspective. Thirty-three patients aged 12-17 years with confirmed or suspected ADPKD participated in semi-structured interviews to elicit their experiences of symptoms and physical, social, and emotional impacts. Interviews were conducted in 13 countries. Substantial proportions of adolescents reported symptoms, including pain (64%), abnormal feelings of fullness (30%), and urinary symptoms (52%) such as urinary frequency and urgency. Thirty percent of participants reported limiting their participation in sports, 21% were bothered by dietary limitations, and 18% had missed school. Although ADPKD is commonly understood as an adult disease, greater awareness of the disease burden in young patients with ADPKD is needed.

Autosomal dominant polycystic kidney disease (ADPKD) is the most common inherited kidney disorder.^1^^,^^2^ It is a progressive, systemic disease characterized by the formation and growth of renal cysts, enlargement of the kidneys, destruction of renal parenchyma, and reduced kidney function, which eventually progresses to kidney failure in adulthood.^1^ The disease also affects other organ systems, including the liver, heart, connective tissue, gastrointestinal system, and cerebral vasculature.^3^

The prevalence of ADPKD is estimated to be 3.96/10,000 population.^4^ The average age of diagnosis in the United States is about 30 years, but the disease presents with a wide clinical spectrum, starting as early as in utero or infancy. The disease typically progresses slowly, with a long latency period. Most clinical symptoms do not appear until the third or fourth decade of life.^5^ Children and adolescents are typically asymptomatic; only 2%-5% of ADPKD patients present in childhood.^5^^,^^6^ Urinary concentrating defects, gross hematuria, abdominal pain, and hypertension are typically some of the earliest clinical manifestations in the pediatric population.^1^^,^^6^^,^^7^ Treatment includes hypertension control, management of cyst-related complications (eg, abdominal pain, urinary tract infections), and dietary restrictions.^8^

Although genetic testing and screening are available, children considered at risk for ADPKD because of family history are not routinely screened.^1^^,^^6^ Clinicians may be hesitant to formally diagnose the disease in children because of legal and ethical issues associated with genetic testing in a vulnerable population. Furthermore, with no approved treatments available for this age group, receiving a diagnosis can have an adverse psychological impact on patients and their families.^9^ A formal diagnosis may impact health, life, and disability insurance options for the rest of the patient’s life, even while the disease may be mostly asymptomatic.^1^^,^^6^ Thus, a diagnosis of ADPKD often occurs incidental to diagnostic imaging tests in childhood, obstetric ultrasound, or through screening in high-risk families. These diagnostic constraints make it challenging to conduct research in children and adolescents with ADPKD.^10^

Some research has found that adults with early-stage disease experience substantial physical symptoms and emotional effects and that physicians tend to underestimate patients’ symptoms and underappreciate the disease’s impacts until patients are close to kidney failure.^5^^,^^11^ Even less is known about the disease’s impact in the pediatric population. Yet, the long duration and progressive nature of ADPKD, the likely presence in the home of a parent or other family member with the disease, the unique emotional and social needs of adolescents, and the possible underrecognition of symptoms by physicians suggest that a better understanding of disease impact in this age group is needed.^10^

The objective of this qualitative study was to explore the burden of disease in adolescents with ADPKD, including the symptoms, impact on daily life, and emotional and social issues and concerns.

## Methods

In this study, individual interviews were conducted with adolescent boys and girls in several countries to explore the health-related quality of life issues. The study was conducted within the context of a project to develop a new patient-reported outcomes (PRO) instrument that would assess disease impacts in adolescents with ADPKD. The purpose of this paper is to report qualitative findings on the burden of disease in adolescents, which were obtained during interviews conducted as part of the instrument development process. The methods conformed to recommended guidance concerning the conduct of qualitative research for the development of PRO tools.^12^, ^13^, ^14^ The research team included individuals with expertise in qualitative research, PRO development and validation (D.O., J.C.C., A.C.P.), and pediatric nephrology (F.S., L.G.W.); none of the researchers had any prior association with the participants before the study. The study was reviewed by the New England Institutional Review Board.

Semi-structured interviews were chosen as the method for obtaining qualitative data, as they allow patients’ voices to shape the concepts and terminology and permit in-depth exploration of concepts.^15^ Focus groups were also considered; however, parents or caregivers of potential study participants were reluctant to have their adolescents participate in a group setting and were more amenable to a 1-on-1, semi-structured interview format. An interview guide was developed based on our prior findings on ADPKD impacts in adults, a review of the literature concerning ADPKD in pediatric populations, and consultation with pediatric nephrologists.^11^ The goal of the interview was to elicit information about disease history, ADPKD-related symptoms (including their frequency and intensity), and the impacts of the disease on the participant. Impacts of interest included physical, social, and emotional impacts in the past and at the time of the interview, as well as the perceived impact of the disease on future life plans (eg, career goals, family plans).

### Participants

Participants were recruited in 13 countries (18 cities) in Asia, Europe, North America, and South America. Eligible participants were: (1) 12-17 years old at the time of the interview; and (2) had a diagnosis of ADPKD or were under the care of a physician for suspected ADPKD in the absence of a confirmed diagnosis. Validated diagnostic criteria for ADPKD in the pediatric population are lacking, and many families are reluctant to pursue a formal diagnosis before adulthood, given the absence of an approved treatment and the potential impact of a diagnosis on insurability in some countries.^8^ Accordingly, a statement by a clinician that the potential participant was suspected to have ADPKD in the absence of a formal diagnosis was sufficient to meet the second eligibility criterion, whereas confirmation by a parent that the child had ADPKD was insufficient.

A market research institute recruited participants through pediatric nephrologists, health foundations, and public advertisements. Interested families with potentially eligible children were screened for eligibility via telephone. The parent or legal guardian of the eligible participating adolescent provided signed informed consent. In addition, the participating adolescent signed an assent form to document their understanding and agreement with the research. Given the difficulties in diagnosing and recruiting pediatric patients with ADPKD as described above, no sample size calculation was conducted beyond the goal of obtaining up to 6 participants per study site.

### Data Collection

Data collection took place between 2012 and 2013 at the market research facility. Demographic data and medical history information were collected from the participant and via the parent or guardian. Semi-structured interviews were conducted by interviewers employed by Covance Market Access Service who were trained in qualitative research methods and had experience in performing pediatric research. The interviews were held in the participant’s native language using the interview guide and were video recorded to aid transcription. In countries where English was the native language, the Covance staff or designee moderated the individual interviews in person. For interviews conducted in a language other than English, simultaneous translation was provided for the observing study staff. Each interview room had a 1-way mirror through which study personnel in an adjoining room could observe the process. To protect patient confidentiality, only the first name of the participant was used during the interview.

### Analysis

The interviews were transcribed and coded to identify conceptual themes using HyperRESEARCH qualitative research software (ResearchWare, Inc). Themes were derived in an iterative process, in which a single coder (A.C.P.) generated codes based on feedback from the interview participants and tracked confirmations and new concepts as they arose across subsequent interviews. All data were deidentified to protect patient confidentiality. A table of the ADPKD-related symptoms and the impacts reported and endorsed by the participants was developed to determine data saturation (the point at which no new concepts or themes were identified during subsequent interviews).^16^ The data were reviewed and organized into related concepts. Outputs were reviewed during cognitive debriefing interviews with a second group of participants to discuss the understandability, readability, and clarity of a draft questionnaire developed from the concept elicitation interviews. The eligibility criteria for the participants in the cognitive debriefing interviews were the same as those for the participants in the concept elicitation interviews. Participants reviewed the draft instrument and were asked questions about the instructions, wording, questions, and response options and how they might be improved.

## Results

**Figure 1:**
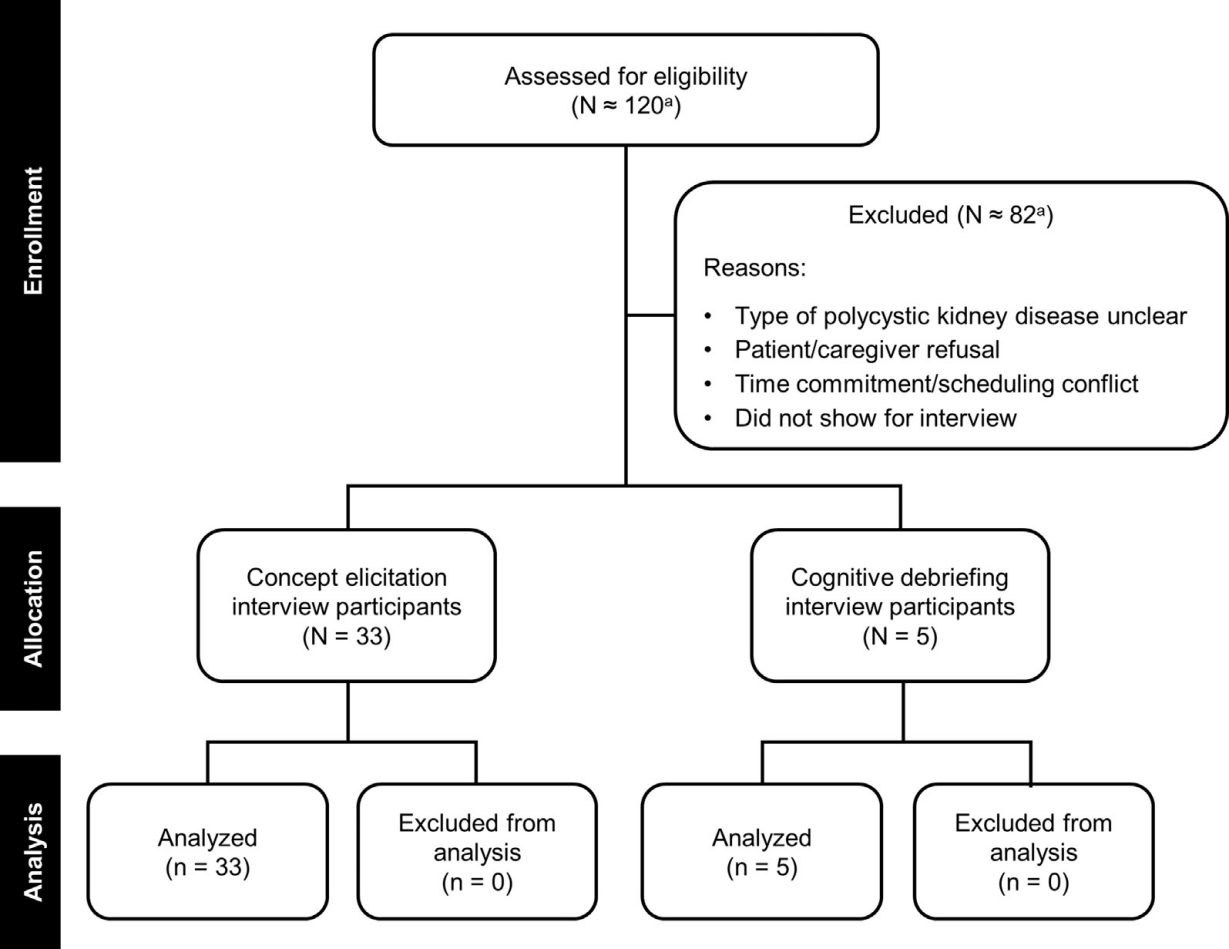
Flowchart of patient numbers at each study stage. ^a^The total number of individuals screened was not recorded at some study sites.

Local market research institute offices contacted the families of approximately 120 adolescents about participating in the interviews and screened them for interest and eligibility. Of this group, 33 adolescents in 13 countries participated in the interviews (Table 1 and Fig 1). Reasons for exclusion included an unclear type of polycystic kidney disease, parent or caregiver refusal, the time commitment or scheduling conflicts, and not appearing for the scheduled interview. Participants had a mean age of 14.6 years, 42.4% were female, and 90.9% indicated a family history of ADPKD (Table 2). Parent or caregiver refusal was most often due to worry that the interview process would scare their child and show aspects of the disease that parents had not previously shared with their child. Adolescents who participated in the research had often researched the disease on the internet and had a much better understanding of the disease than the parent or caregiver assumed they had. Adolescents also reported a reluctance to discuss their symptoms with the parent or caregiver to avoid causing worry and because of a perception that the parents were already too burdened by their own ADPKD or caregiving responsibilities for another family member.

**Table 1:** Location of Interviews and Number of Participants

Country	City	No. of Participants (N = 33)
Argentina	Buenos Aires	2
Brazil	São Paolo	2
Canada	Toronto	1
Czech Republic	Prague	2
Germany	Berlin	3
	Heidelberg	2
Hungary	Budapest	3
Japan	Tokyo	2
Poland	Warsaw	1
Romania	Bucharest	2
Spain	Madrid	2
Taiwan	Taipei	2
United Kingdom	London	2
United States	Alexandria, Virginia	1
	Atlanta, Georgia	1
	Boston, Massachusetts	2
	Denver, Colorado	2
	Los Angeles, California	1

**Table 2:** Demographic and Clinical Characteristics of Adolescents with ADPKD Participating in the Interviews

Characteristic	Participants (N = 33)
Age, mean (SD)	14.6 (1.8)
Gender, n (%)	
Male	19 (57.6%)
Female	14 (42.4%)
Family history, n (%)	30 (90.9%)

### Symptom Reports

The most frequently reported symptoms were pain and urinary issues, with no major differences between girls and boys in symptom frequency. Twenty-one participants (63.6%) reported some type of pain attributed to their ADPKD; more than one-quarter of participants reported an ache, hurt, or pain in the back (n = 9; 27.3%), and nearly one-fifth (n = 6; 18.2%) reported kidney pain (Table 3). Some participants reported discomfort, dull pain, or a feeling of “pressure” or “heavy kidneys.” Others reported pain that was gnawing, throbbing, pulsating, sudden, cramping, sharp, stabbing, or stinging. One participant said, “It is just a sudden pain and it’s this penetrating pain” (Table 4)*.* Another said, “I was just sitting and I felt the pain.” Although most of the pain was described as mild to moderate, intense pain due to rupture of a cyst or infection was reported by 5 participants (15.2%). One participant said, “They [kidneys] hurt so bad I could not get out of bed.” The pain frequency ranged from seldom (eg, 1 or 2 episodes in a 6-month period) to daily. Most pain episodes were short, lasting from seconds to minutes, although some episodes lasted 15 minutes or longer. Three participants (9.1%) complained of headaches attributed to ADPKD-related hypertension.

**Table 3:** Frequency of Symptoms Reported by Adolescents with ADPKD

Symptom	No. (%) of All Participants (N = 33)	No. (%) of Females (n = 14)	No. (%) of Males (n = 19)
No symptoms	5 (15.2%)	1 (7.1%)	4 (21.1%)
Hypertension	11 (33.3%)	5 (35.7%)	6 (31.6%)
Hematuria	4 (12.1%)	1 (7.1%)	3 (15.8%)
Abdomen shape and size	3 (9.1%)	2 (14.3%)	1 (5.3%)
Bulge or “weirdly” shaped	2 (6.1%)	1 (7.1%)	1 (5.3%)
Can feel kidneys	1 (3.0%)	1 (7.1%)	0
Can’t bend over	1 (3.0%)	1 (7.1%)	0
Pain	21 (63.6%)	11 (78.6%)	10 (52.6%)
Ache or hurt or pain in back	9 (27.3%)	6 (42.9%)	3 (15.8%)
Kidney(s) hurt or pain	6 (18.2%)	3 (21.4%)	3 (15.8%)
Uncomfortable or discomfort	6 (18.2%)	3 (21.4%)	3 (15.8%)
Intense pain (burst or infection)	5 (15.2%)	3 (21.4%)	2 (10.5%)
Pain in front, stomach	4 (12.1%)	2 (14.3%)	2 (10.5%)
Cramp or stitch (pain)	3 (9.1%)	1 (7.1%)	2 (10.5%)
Dull pain	3 (9.1%)	1 (7.1%)	2 (10.5%)
Headache	3 (9.1%)	1 (7.1%)	2 (10.5%)
Kidneys hurt during or after urination	3 (9.1%)	2 (14.3%)	1 (5.3%)
Flank pain or pain in the sides	2 (6.1%)	2 (14.3%)	0
“Pain” (not otherwise identified)	2 (6.1%)	1 (7.1%)	1 (5.3%)
“Pressure”	2 (6.1%)	1 (7.1%)	1 (5.3%)
“Sharp” or “stabbing” pain	2 (6.1%)	1 (7.1%)	1 (5.3%)
Sudden pain	2 (6.1%)	1 (7.1%)	1 (5.3%)
“Gnawing, episodic”	1 (3.0%)	1 (7.1%)	0
“Heavy” kidneys	1 (3.0%)	0	1 (5.3%)
Pulsating pain	1 (3.0%)	0	1 (5.3%)
“Stinging” pain	1 (3.0%)	1 (7.1%)	0
Throbbing pain	1 (3.0%)	1 (7.1%)	0
Fullness	10 (30.3%)	5 (35.7%)	5 (26.3%)
Stomach felt full	6 (18.2%)	3 (21.4%)	3 (15.8%)
Blocked sensation	1 (3.0%)	1 (7.1%)	0
Not hungry	1 (3.0%)	1 (7.1%)	0
Unclear terminology	2 (6.1%)	0	2 (10.5%)
Urinary frequency and urgency	17 (51.5%)	8 (57.1%)	9 (47.4%)
Urinary urgency	10 (30.3%)	4 (28.6%)	6 (31.6%)
Urinary frequency	6 (18.2%)	3 (21.4%)	3 (15.8%)
Because I drink more water	6 (18.2%)	4 (28.6%)	2 (10.5%)
Urinate at night	3 (9.1%)	1 (7.1%)	2 (10.5%)

**Table 4:** Sample Quotes from Adolescents About ADPKD Symptoms and Impacts

Symptom or Impact	Examples of Participants' Comments
Hypertension	“Like if like you stand up you get like really dizzy and see a bunch of different colors for a couple of seconds.” “If I don’t take my pills I will get really light-headed easily and then so like when I go to [amusement park] or something like that I can’t ride too many of the rides because I get really light-headed just going up and down.”
Pain characteristics	“Gnawing pain in the head and back.” “At the end of peeing my kidneys hurts a little bit.” “It stings when I pee.” “One or both sides as well, at the back.” “Pinprick sensation in the kidneys.”
Pain intensity	“They [kidneys] hurt so bad I could not get out of bed.” “I can’t stand straight anymore; my lower back hurts a lot.” “This pain is dull. It makes me feel numb when it is very strong, pulsating in intensity.” “It’s really sharp and like literally it feels like I’m being stabbed ….”
Pain attribution and duration	“When I am in a bad position for a long time.” “It can last from hours to minutes. Sometimes it’s like a contraction thing, like 5 minutes really, really sharp, and then I get a 10-minute break where it’s just dull and then it’s on and off like that.” “I’ve had cyst ruptures that literally keep me down for 20 minutes and then I’m fine.”
Pain impact on daily activities	“I can’t practice sports; I am not allowed at all, if I do my knees hurt a lot, I can’t feel my legs, and I start to tremble. I get chills and so on, and that’s that, and my kidneys hurt.” “No, I can do everything. It’s simply there. I think I’ve gotten used to it. It may put me in a bad mood.” “When the pain is bad I lay in bed and I don’t feel like doing anything.”
Pain frequency	“Maybe once in 5 months.” “Once a week more or less.” “A throbbing pain all the time.” “Bad pain happens at longer intervals.”
Fullness	“There is something that … is blocking after 2 bites.”
Urinary urgency and frequency	“All of the sudden I just have to go.” “This is sudden, that’s every day.” “I go to the bathroom and maybe 10 or 15 minutes later I have to go again.”
General impact on daily activities	“If I go by what physicians say I wouldn’t be allowed to do anything. I do sometime. I also need to have fun.”
Impact on sports or physical activity	“I need to use my body sparingly. For example, I try to avoid running, because I am afraid it will affect my health.”
Impacts on school	“It takes a lot out on a kid and like, I don’t know basic because none of the teachers wanted to teach me after I got back… And it has a really big effect on children because their learning is really affected by it and that’s probably my biggest concern.” “When I’ve had like a bladder infection, I need to go to the toilet and therefore I’m missing out …”
Dietary impacts	“A key thing is food restriction, and I also drink a lot of water.” “I have to watch my salt intake. I have to avoid chips, those things that are really salty.” “… all my friends are eating chips and popcorn and I’m just like eating an apple.”
Impacts on social activities	“I have to stop and think, I cannot do this right now because of this or because I have this condition.” “… I was not able to go on the rides because of my back …
Emotional impacts	“It either freaks me out if I keep thinking about it every day …” “You can’t really talk about it with anybody.” “When we talk about it I usually feel sad.” “When I first learned about it, I was pretty angry.”
Impact on life plans	“I want to have children, and pregnancy can be a problem and I know it’s a hereditary disease, so I will have to think about my children, whether I want to take the risk.” “Knowing that my kids may have the predisposition to some disease makes me sad.” “I will probably not consider having kids. I may adopt kids because I am afraid of passing it onto my children.”

Nearly one-third of participants (n = 10; 30.3%) reported a symptom of fullness. Participants described this as feeling full quickly when eating, not feeling hungry at all, or feeling full despite not having eaten in a long time. One participant described the feeling of fullness as, “There is something that… is blocking after 2 bites.” About half of the participants reporting fullness experienced their symptoms daily or several times a week.

Seventeen participants (51.5%) reported some type of urinary symptom, with urinary urgency being the most common (n = 10; 30.3%). This was typically described as a sudden need to “pee.” One participant described this as, “All of the sudden, I just have to go.” Some participants experienced urinary urgency on a daily basis. Three participants (9.1%) reported that it interrupted their sleep, with the number of awakenings ranging from 1 or 2 to more than 5 times per night. Six participants attributed their urinary symptoms to drinking more water because they had been instructed to do so by their doctor or parent to aid kidney health, whereas the other participants with urinary symptoms reported them without attribution to increased fluid intake. Five participants (15.2%) reported no ADPKD-related symptoms.

### Impact Reports

The disease impacts reported by participants are shown in Table 5. Seven participants (21.2%) reported no impact of ADPKD on their daily life. Ten participants (30.3%) reported that they avoided sports based on the advice of their doctor or parent because they experienced pain or discomfort during physical activity, or for other reasons. In addition, 6 participants (18.2%) indicated that they had missed school because of ADPKD. Some participants also mentioned feeling uncomfortable at school because of the need to urinate frequently. About one-fifth (n = 7; 21.2%) of participants reported that they were bothered or impacted by dietary limitations (primarily the need for reduced sodium intake and increased water intake). One participant said, “I have to watch my salt intake, I have to avoid chips, those things that are really salty.”

**Table 5:** Frequency of Disease Impacts Reported by Adolescents with ADPKD

Impacts^a^	No. (%) of All Participants (N = 33)	No. (%) of Females (n = 14)	No. (%) of Males (n = 19)
No impacts	7 (21.2%)	3 (21.4%)	4 (21.1%)
Tiredness			
“Tired” or “fatigued”	2 (6.1%)	1 (7.1%)	1 (5.3%)
No strength	1 (3.0%)	1 (7.1%)	0
No energy (to do things)	1 (3.0%)	1 (7.1%)	0
Exhausted at the end of the day	1 (3.0%)	0	1 (5.3%)
Impacts on activities			
Can’t do sports or avoid sports	10 (30.3%)	6 (42.9%)	4 (21.1%)
Missed, skipped a class, or left school	6 (18.2%)	1 (7.1%)	5 (26.3%)
Felt uncomfortable at school	2 (6.1%)	1 (7.1%)	1 (5.3%)
Delay doing things	2 (6.1%)	0	2 (10.5%)
Causes difficulties at work	2 (6.1%)	1 (7.1%)	1 (5.3%)
Sometimes do not listen to doctor	1 (3.0%)	1 (7.1%)	0
Dietary impacts			
Have to watch salt intake or drink more water	7 (21.2%)	1 (7.1%)	6 (31.6%)
Social impacts			
Cannot do something with friends	6 (18.2%)	3 (21.4%)	3 (15.8%)
Do not talk with friends about it	3 (9.1%)	2 (14.3%)	1 (5.3%)
Compare self to others	2 (6.1%)	1 (7.1%)	1 (5.3%)
Trust friends (not to gossip)	1 (3.0%)	0	1 (5.3%)
Emotional impacts			
Worried (about disease and future)	6 (18.2%)	2 (14.3%)	4 (21.1%)
Accept disease (as is)	5 (15.2%)	3 (21.4%)	2 (10.5%)
Feel sad	4 (12.1%)	1 (7.1%)	3 (15.8%)
Do not want to think about PKD	4 (12.1%)	3 (21.4%)	1 (5.3%)
Frustrated	3 (9.1%)	1 (7.1%)	2 (10.5%)
Annoyed by PKD symptoms	1 (3.0%)	0	1 (5.3%)
Nervous	1 (3.0%)	1 (7.1%)	0
Life-plan impacts			
Having children	7 (21.2%)	3 (21.4%)	4 (21.1%)
Changed career path	1 (3.0%)	1 (7.1%)	0
Difficult to get a job	1 (3.0%)	0	1 (5.3%)

Abbreviation: PKD, polycystic kidney disease.

a Study participants’ wording or phrasing has been retained when listing some impacts.

Social impacts were also evident. Six participants (18.2%) said they were not able to or did not engage in some activities with friends; this was related to the desire to keep their condition secret, avoid being teased, or avoid the feeling of being different. One participant said, “You can’t really talk about it with anybody.” In terms of emotional impacts, most participants reported that they had adapted to the fact that they had a progressive, chronic disease. Some expressed the desire to see themselves as normal and said they did not want to think about having the disease.

Between 3.0% (n = 1) and 18.2% (n = 6) of participants reported feeling nervous, frustrated, sad, or worried about the disease and their future. These participants referred to having problems accepting their diagnosis, its impacts, and the restrictions that come with the disease. Many worried that their kidney health would deteriorate and indicated that they were afraid of the consequences. For example, 1 participant said, “My kidneys will not last that long.” Seven participants (21.2%) expressed concern about having children in the future because of the hereditary risk for ADPKD. Although participants generally did not experience emotional concerns on a daily basis, some reported that receiving medical checkups or other medical care renewed their feelings of worry or concern about their condition.

### Cognitive Debriefing Interviews

Five individuals participated in cognitive debriefing interviews conducted in London, Manchester, and Huddersfield, United Kingdom. The mean age was 13.6 years, 40% were female, 100% were White, and 80% indicated a family history of ADPKD. The participants confirmed the concepts elicited in the concept elicitation interviews, including concepts related to pain, tiredness, urinary frequency and urgency, impacts on daily activities, and emotional impacts.

## Discussion

This qualitative, observational study of adolescents with ADPKD in 13 countries found that although most adolescents experience little or no disease burden, a substantial proportion experience a variety of physical, social, and emotional impacts. Pain—ranging from mild to intense—was a frequently reported symptom. Pain in the back was experienced by nearly 3 in 10 participants, and pain characterized as kidney pain was reported by nearly 2 in 10 participants. Urinary symptoms were also common; nearly one-third of adolescents reported urinary urgency, and nearly one-fifth reported frequent urination. A substantial proportion of adolescents experienced social impacts, with nearly one-fifth reporting that they had missed school or not participated in social activities and nearly one-third saying that they avoided sports and physical activity because of pain or concern that this would make their condition worse. Approximately one-fifth of adolescents indicated that they needed to manage their diet, primarily by limiting sodium intake and ensuring adequate water consumption. Some adolescents reported emotional impacts as well; nearly one-fifth cited worry about their health and future, and others mentioned feelings of sadness, isolation, and frustration.

These findings counter the common perception that most adolescents with ADPKD do not experience pain or discomfort from their disease. The findings accord with a recent study that found that 88% of adults with early-stage ADPKD experienced back, kidney, or abdominal pain, as well as general malaise, fatigue, and breathlessness.^5^ That study also found that emotions of loss, uncertainty, and fear were common, and that physicians underestimate both the physical symptoms and emotional impacts experienced by patients with early-stage disease. Interestingly, a retrospective cohort study found that ADPKD patients with normal kidney function had substantially greater use of health care services and higher costs relative to the general population.^17^ Our findings regarding pain characteristics in adolescents are also in agreement with findings from recent studies of adults with ADPKD.^11^^,^^18^ That research revealed that individuals with ADPKD experience 3 primary types of pain: dull kidney pain, severe or sharp kidney pain, and a feeling of fullness and/or discomfort. These types of pain also were observed in the present study. The urinary urgency, frequency, and nocturia reported by adolescents in this study have all been identified as symptoms in adults.^11^ Of the 17 participants in this study who reported urinary symptoms, approximately one-third (n = 6) attributed them to increased water intake to slow disease progression. The other participants with urinary symptoms did not make this connection; in these cases, it seems likely that the symptoms may have been due to ADPKD-related urinary concentrating deficits and associated increases in thirst.

This exploratory study provides new information about disease impacts in adolescents with ADPKD and points to the need for more comprehensive research. Such research may help clinicians to better understand the disease burden of ADPKD in adolescents and help them cope with health-related quality of life issues, including pain, discomfort, and social and emotional concerns. The results of this study also add to the general discussion concerning the potential benefits and hazards of diagnosing ADPKD in adolescents. It is possible that an earlier formal diagnosis and appropriate care could result in better pain management, improved psychological well-being, and the adoption of lifestyle habits that improve health and contribute to optimal disease management. The investigators intend to use the findings from this qualitative research to guide further development of a PRO tool that can be used to assess health-related quality of life issues in adolescents with ADPKD. In addition, a future publication will report additional data obtained during this project related to parents and other caregivers of children with ADPKD.

The study has some limitations. The sample size was relatively small because of the difficulty, despite repeated efforts, in recruiting adolescents for the study. It is also possible that selection bias occurred. Adolescents from families with a history of more advanced or aggressive ADPKD were possibly more likely to participate because they might be more likely to recognize symptoms and might have more to share about the burden of disease. In comparison, adolescents from families with less awareness of symptoms might have decided not to participate since they felt they did not have enough information to discuss in the study, or the parents might not have wanted to expose their child to additional information about the disease. An additional limitation is that we were not able to conduct focus groups with participants because of a lack of parental or guardian consent. Conducting focus groups might have yielded additional information because participants in groups are exposed to other participants' comments, possibly leading to additional elicited concepts. Nonetheless, the carefully developed interview guide and the comprehensive interviews that were conducted yielded information and specific details that might not have been revealed in a group setting. A significant strength of the study is its geographic diversity: it included participants from countries in North America, South America, Asia, and Europe. Because of this diversity, the findings may be generalizable to the larger adolescent ADPKD population.

In conclusion, adolescents with ADPKD exhibit a wide diversity of symptoms and disease impacts. Contrary to common perception, a substantial proportion of adolescents with ADPKD experience pain and urinary symptoms, as well as social and emotional impacts. Overall, the experience of the disease and its symptoms was very similar to that of adult patients, indicating that the impact of ADPKD is felt earlier than commonly thought to be the case, especially in adolescents with rapidly progressing disease. These qualitative findings provide a foundation for further research and may help the medical community to better address the health and emotional needs of adolescents with ADPKD, who have a lifetime of challenges ahead of them.
